# CD109 is a novel marker for squamous cell/adenosquamous carcinomas of the gallbladder

**DOI:** 10.1186/s13000-015-0375-0

**Published:** 2015-08-07

**Authors:** Fengyun Dong, Chuanfeng Lu, Xiaocui Chen, Yuan Guo, Ju Liu

**Affiliations:** Medical Research Center, Shandong Provincial Qianfoshan Hospital, Shandong University, 16766 Jingshi Road, Jinan, Shandong 250014 China

## Abstract

Gallbladder cancer is the most common biliary tract malignancy with the worst overall prognosis. CD109 is a co-receptor of TGF-β1 and suppresses TGF-β signaling. In this study, CD109 protein expression in three subtypes of gallbladder cancer was examined by immunohistochemistry on human tissue samples and tissue microarrays. We found that CD109 is specifically expressed in malignant squamous cells in squamous cell carcinomas (86.7 %) and adenosquamous carcinomas (91.7 %), but not in adenocarcinomas or normal gallbladder tissues. Thus, CD109 may be a potential pathology marker for gallbladder squamous cell/adenosquamous carcinomas.

## Findings

Gallbladder cancer (GBC), the most common malignancy of the biliary tract, is a highly lethal disease with an overall 5 year survival of less than 5 % and mean survival of 6 months [1, 2]. GBC belongs to the epithelium originated tumor of the digestive system with a highly variable presentation [2]. More than 80 % of GBCs are adenocarcinomas (AC) arising from the gallbladder mucosa. Squamous cell carcinoma (SCC), adenosquamous carcinoma (ASC), undifferentiated or anaplastic carcinoma represent less common types of GBCs [3]. Up to date, no biological markers for effectively identifying GBC subtypes have been reported. CD109, a glycosyl-phosphatidylinositol anchored protein, is a member of the a2-macroglobulin/complement family. CD109 binds transforming growth factor (TGF)-β1 with high affinity, and suppresses TGF-β signaling [4]. In mammals, CD109 is highly expressed in squamous cells of skin, uterus and esophagus [5–7], but has never been examined in GBC tissues. In this study, we determined CD109 expression in 3 subtypes of GBC by immunohistochemistry.

This study was performed in accordance with the “Code for Proper Secondary Use of Human Tissue”. The specimens were obtained from Pathology Center at Shandong Provincial Qianfoshan Hospital (Jinan, China) and the tissue microarrays (TMAs) from US Biomax Inc (GA801, Rockville, MD). In total, we collected the sections of 21 gallbladder ACs, 16 SCCs, 12 ASCs and 10 normal gallbladder tissues. Immunohistochemical staining was performed using a rabbit polyclonal anti-CD109 antibody (HPA009292; Sigma-Aldrich, St Louis, MO) with appropriate controls. The experiments were repeated to confirm reproducibility. After staining, the tissue sections and TMAs were photographed using an OLYMPUS FSX100 imaging system (Olympus Corporation, Tokyo, Japan). The data were analyzed by Pearson's chi-squared test using SPSS software (SPSS Inc, Chicago, IL).

**Fig. 1:**
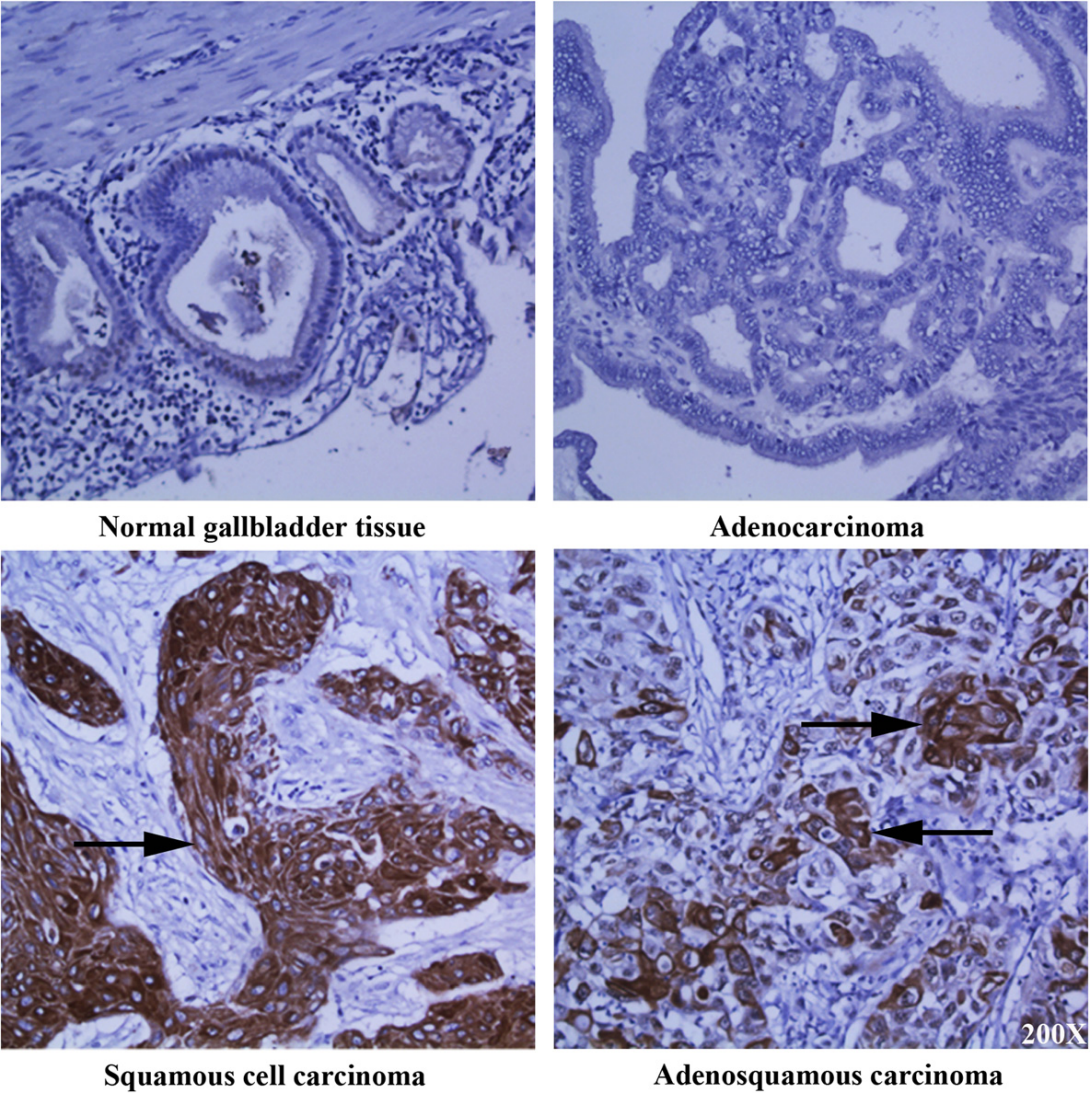
CD109 expression is restricted in squamous cell/adenosquamous carcinomas of the gallbladder. Immunohistochemistry of CD109 on normal gallbladder tissue, adenocarcinoma, squamous cell carcinoma, and adenosquamous carcinoma of the gallbladder. Arrows refer to CD109 positive cells. Magnification: 200X

As shown on Table 1, CD109 staining was negative in all normal gallbladder tissues and AC tissues. CD109 positive cells were found in 86.7 % of SCCs and 91.7 % of ASCs. On the sections of SCCs and ASCs, CD109 staining is restricted in the membrane and cytosol of malignant squamous cells, which are stratified, disorganized and invaded into the submucosa layer (Fig. 1, C,D). For tumor grade, the percentage of CD109 positive cases are similar in well- (100 %) and moderately- (83.3 %) differentiated SCCs (p = 0.26). In addition, the level of CD109 expression showed no significant difference in well- and moderately-differentiated SCCs (percentage of expression: 24.85 ± 3.48 vs. 18.64 ± 4.62, p = 0.14;intensity of cytoplasmic staining: 0.36 ± 0.02 vs. 0.37 ± 0.04, p = 0.42). CD 109 is not expressed in other cell types, including column epithelial cells, fibroblast, endothelial cells, and smooth muscle cells in all the normal and tumor tissues (Fig. 1).

**Table 1:** Summary of CD109 expression in normal gallbladder tissues and subtypes of gallbladder cancer

Tissue sample	n	CD109 expression		*P* value
		Negative (n, %)	Positive (n, %)	
Normal gallbladder tissues	10	10 (100)	0 (0)	
Adenocarcinoma	19	19 (100)	0 (0)	
Squamous cell carcinoma	15	2 (13.3)	13 (86.7)	<0.001*
Adenosquamous carcinoma	12	1 (8.3)	11 (91.7)	<0.001**

*Significantly different CD109 expression between squamous cell carcinoma and normal gallbladder tissues

**Significantly different CD109 expression between adenosquamous carcinoma and normal gallbladder tissues

SCCs and ASCs account for an estimated 1.4–10.6 % of all incidences of gallbladder carcinoma [8]. The clinicopathological features of SCC/ASCs and the differences from ACs, the major subtype of GBC, have rarely been described [9]. On regular examination, approximately 10-37 % of the GBCs cannot be identified with certainty [10]. SCCs of the gallbladder might have a poorer prognosis than ACs, and the accurate diagnosis is crucial to ensure the most effective treatment after surgery. To our knowledge, this is the first report that CD109 protein expression is specifically expressed in malignant squamous cells of gallbladder SCC and ASC tissues. Higher level expression of the CD109 gene and/or protein was also detected in SCCs of other organs [5–7]. Therefore, CD109 may be a potential marker for gallbladder SCCs and ASCs. In the skin, p63 has been used as a marker of SCC, but also labels basal cell carcinomas [11]. The p40 antibody recognizes the isoform of the p63 protein without the transactivation domain (the ΔNp63 isoforms) [12]. The p63 showed focal cytoplasmic staining in addition to nuclear staining, while exclusive nuclear staining is observed with p40 with no cytoplasmic reactivity in SCCs [13]. To date the antibodies of p63 and p40 have not been tested for gallbladder cancers. Unlike p63 or p40, CD109 expression is exclusively in the membrane and cytoplasma with no staining in nucleus of SCCs, thus representing a different type of pathology marker.

The molecular mechanisms underlying the genesis and progression and of GBCs are still unclear. The squamous cell component of the gallbladder was reported to have greater proliferation capacity than the glandular component [14, 15]. TGF-β signaling maintains epithelial homeostasis [16], and defective TGF-β signaling causes hyperproliferation, reduced apoptosis and increased genomic instability in squamous cells [17]. Reduced expression of TGF-β signaling components is commonly observed in human SCCs [18]. CD109 is a co-receptor for TGF-β1 and increases the binding of TGF-β1 to its receptors [4]. CD109 associates with caveolin-1, a major component of the caveolae, and facilitates localization of the TGF-β receptors into the caveolar compartment where they degrade [19]. Thus, CD109 enhances TGF-β receptor endocytosis and negatively regulates TGF-β signaling. Elevated expression of CD109 in gallbladder squamous cells may inhibit TGF-β signaling and subsequently promote the development of SCC. Taken together, CD109 may involve in the pathogenesis of gallbladder SCCs and present as a novel target for therapeutic intervention.

## Abbreviations

GBC: Gallbladder cancer
AC: adenocarcinomas
SCC: Squamous cell carcinoma
ASC: adenosquamous carcinoma
TMA: tissue microarrays
TGF-β: Transforming growth factor –β

## References

[1] Lai CH, Lau WY (2008). Gallbladder cancer--a comprehensive review. Surgeon.

[2] Wernberg JA, Lucarelli DD (2014). Gallbladder cancer. Surg Clin North Am.

[3] Gourgiotis S, Kocher HM, Solaini L, Yarollahi A, Tsiambas E, Salemis NS (2008). Gallbladder cancer. Am J Surg.

[4] Finnson KW, Tam BY, Liu K, Marcoux A, Lepage P, Roy S (2006). Identification of CD109 as part of the TGF-beta receptor system in human keratinocytes. FASEB J.

[5] Dong F, Wang Y, Li L, Wang Y, Liu X, Liu J (2014). CD109 expression is increased in cutaneous squamous cell carcinoma. J Dermatol.

[6] Zhang JM, Hashimoto M, Kawai K, Murakumo Y, Sato T, Ichihara M (2005). CD109 expression in squamous cell carcinoma of the uterine cervix. Pathol Int.

[7] Dong F, Liu F, Yan S, Liu X, Jiang Z, Liu J. Elevated Expression of CD109 in Esophageal Squamous Cell Carcinoma. Pathology oncology research : POR. 2015. doi:10.1007/s12253-014-9894-3. 10.1007/s12253-014-9894-3 26122747

[8] Chan KM, Yu MC, Lee WC, Jan YY, Chen MF (2007). Adenosquamous/squamous cell carcinoma of the gallbladder. J Surg Oncol.

[9] Kim WS, Jang KT, Choi DW, Choi SH, Heo JS, You DD (2011). Clinicopathologic analysis of adenosquamous/squamous cell carcinoma of the gallbladder. J Surg Oncol.

[10] Roa I, Araya JC, Villaseca M, Roa J, de Aretxabala X, Ibacache G (1999). Gallbladder cancer in a high risk area: morphological features and spread patterns. Hepatogastroenterology.

[11] Reis-Filho JS, Torio B, Albergaria A, Schmitt FC (2002). p63 expression in normal skin and usual cutaneous carcinomas. J Cutan Pathol.

[12] Henderson SA, Torres-Cabala CA, Curry JL, Bassett RL, Ivan D, Prieto VG (2014). p40 is more specific than p63 for the distinction of atypical fibroxanthoma from other cutaneous spindle cell malignancies. Am J Surg Pathol.

[13] Alomari AK, Glusac EJ, McNiff JM (2014). p40 is a more specific marker than p63 for cutaneous poorly differentiated squamous cell carcinoma. J Cutan Pathol.

[14] Charbit A, Malaise EP, Tubiana M (1971). Relation between the pathological nature and the growth rate of human tumors. Eur J Cancer.

[15] Nishihara K, Nagai E, Izumi Y, Yamaguchi K, Tsuneyoshi M (1994). Adenosquamous carcinoma of the gallbladder: a clinicopathological, immunohistochemical and flow-cytometric study of twenty cases. Japanese journal of cancer research : Gann.

[16] Taylor MA, Lee YH, Schiemann WP (2011). Role of TGF-beta and the tumor microenvironment during mammary tumorigenesis. Gene Expr.

[17] White RA, Malkoski SP, Wang XJ (2010). TGFbeta signaling in head and neck squamous cell carcinoma. Oncogene.

[18] Han G, Wang XJ (2011). Roles of TGFbeta signaling Smads in squamous cell carcinoma. Cell & bioscience.

[19] Bizet AA, Liu K, Tran-Khanh N, Saksena A, Vorstenbosch J, Finnson KW (2011). The TGF-beta co-receptor, CD109, promotes internalization and degradation of TGF-beta receptors. Biochim Biophys Acta.

